# Quantification of gaze reaction time in infants with Pediatric Perimeter

**DOI:** 10.1371/journal.pone.0257459

**Published:** 2021-09-16

**Authors:** Sourav Datta, Koteswararao Chilakala, Sandeep Vempati, Tejopratap Oleti, Jaishree Kulkarni, Srinivas Murki, Pramod Gaddam, PremNandhini Satgunam

**Affiliations:** 1 Brien Holden Institute of Optometry and Vision Science, Hyderabad Eye Research Foundation, L V Prasad Eye Institute, Hyderabad, India; 2 Center for Innovation, Hyderabad Eye Research Foundation, L V Prasad Eye Institute, Hyderabad, India; 3 Fernandez Hospital, Hyderabad, India; Midwestern University, UNITED STATES

## Abstract

**Purpose:**

We quantified the eye/head (gaze) reaction time in infants to establish a normative database for the Pediatric Perimeter device. Additionally, we tested the hypothesis that gaze reaction time will reduce with age.

**Methods:**

A cross-sectional study was conducted. Healthy infants between 3 to 10 months of age were recruited. Peripheral visual field stimuli (hemifield and quadrant stimuli) were presented in the Pediatric Perimeter device. Infant’s gaze to these stimuli was observed, documented in real time, and video recorded for offline analysis.

**Results:**

A total of 121 infants were tested in three age group bins [3–5 months, n = 44; >5–7 months, n = 30 and >7–10 months, n = 47]. Overall, 3–5 months old had longer reaction time when compared to the older infants particularly for stimuli presented in the quadrants (Kruskal-Wallis, p<0.038). A significantly asymmetric difference (p = 0.025) in reaction time was observed between the upper (median = 820ms, IQR = 659-1093ms) and lower quadrants (median = 601ms, IQR = 540-1052ms) only for the 3–5 months old infants.

**Conclusion:**

This study provides the normative gaze reaction time of healthy infants. With increase in age, there is reduction in reaction time and disappearance of reaction time asymmetry in quadrant stimuli. The longer reaction time for upward gaze could be due to delayed maturation of neural mechanisms and/or decreased visual attention.

## Introduction

Pediatric Perimeter is a device that was developed for infants to measure the visual field extent and also to quantify the reaction time taken to gaze (eye and head position) towards a peripheral light stimuli [1]. Pilot data in a previous study [1] showed that a small group of infants with developmental delay (n = 14) were slower in their reaction time by about a factor of two when compared with typically developing infants (n = 5). We wanted to investigate and establish the reaction time in a larger group of typically developing infants. This could then provide a normative database against which comparisons could be made with infants having developmental delay.

In clinical testing, only qualitative and not quantitative observations of infant’s eye/head movement towards a peripheral target are made [2]. Such observations are made to investigate the visual fields or saccadic eye movements. However, developmental research studies have quantified saccadic eye movements. In such studies, reaction time of the saccadic eye movements is known to be longer for younger infants (≤7 months) when compared to older infants [3–5]. The gradual decrease in saccadic reaction time with age suggests that children’s saccades are initiated faster as they grow because of development of the brain saccade circuitry and the anatomical structures of the brain [5]. We therefore hypothesized that a similar trend would also be present when gaze movements (eye and/head movements) are observed in infants when looking towards the peripheral stimuli presented in the Pediatric Perimeter. Thus, a study was undertaken to both test this hypothesis and collect normative data for gaze reaction time in typically developing infants.

## Materials and methods

A cross-sectional study was conducted. The study protocol was reviewed and approved by the Institutional Review Boards of both Fernandez Hospital and L V Prasad Eye Institute. The study protocol followed the tenets of the Declaration of Helsinki. Informed written consent was obtained from the parents after explaining about the research study. Separate written consent was also obtained for video recording and photograph. The individuals in this manuscript have given written informed consent (as outlined in PLOS consent form) to publish these case details.

### Participants

Consecutive healthy infants between 3 to 10 months of age visiting Fernandez Hospital for their vaccination were approached for participation. The data collection was conducted from December 2016 to January 2017. All the recruited infants underwent an examination by the pediatrician and an optometrist before getting enrolled in the study. A detailed birth history consisting of the mode and term of delivery, maternal history for any infections, birth weight of the child, gestation period, history of any incubation, seizures was documented before testing the infants. Only awake infants with an uneventful birth history were recruited. Preterm infants (less than 37 completed weeks of gestation) [6], or low birth weight infants (<2500 grams) [7] or infants with any systemic illness or eye conditions were excluded from the study. Any infant with refractive error of ≥ + 5.00DS hyperopia or ≤ -3.00DS [8] were also excluded. Refractive error was measured using retinoscopy under non-cycloplegic condition. As it was a screening procedure, the exact refractive error was not quantified but merely ensured to see if the infant was within the acceptable refractive error range.

### Pediatric Perimeter

**Fig 1:**
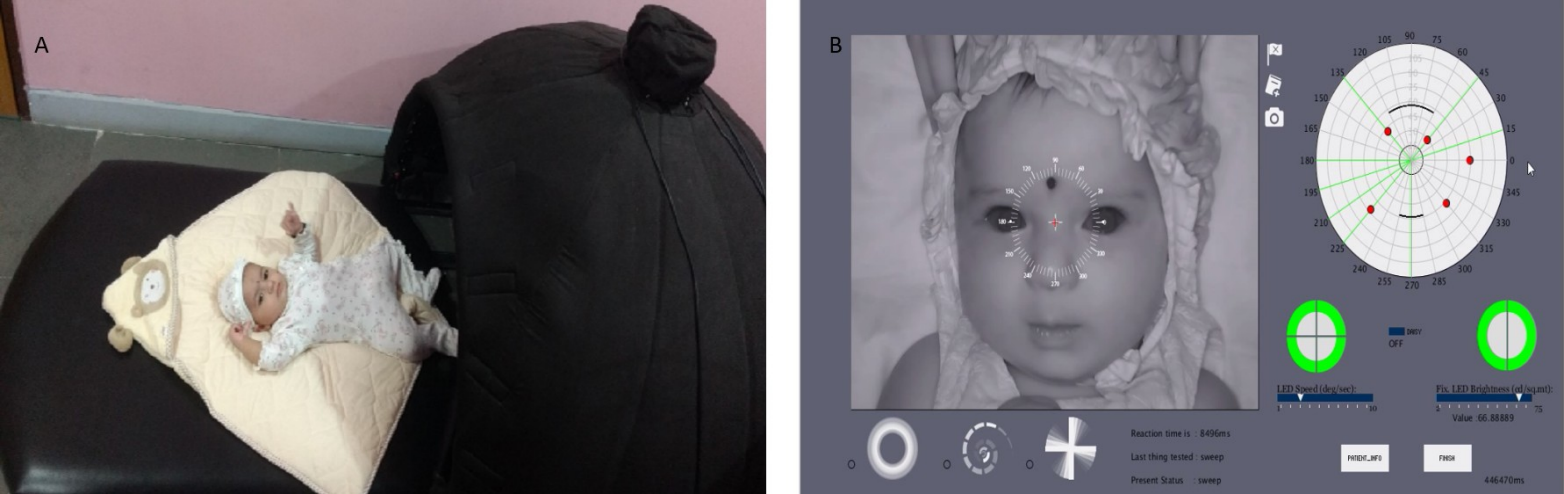
(A) Photograph of an infant before sliding into the Pediatric Perimeter device. (B) Graphical User Interface (GUI) and infrared image capture of the infant’s face is seen. The green colored hemi-circle and quadrants in the lower right represent the stimuli for the Gross Visual Field test.

The Pediatric Perimeter is a dome shaped device built to primarily measure visual fields (Fig 1A). The construction and validation of this device is explained elsewhere [1]. Briefly, the device has 24 meridians at an angular interval of 15° in the azimuthal direction. RGB (red, green and blue) LED (light emitting diode) strips have been mounted on these meridians. The angular distance between two LEDs along a meridian is 3.5° and the luminance of the LED is 30 cd/m^2^ with the presented wavelength matching 550 nm (green-yellow color).

Gross visual field stimuli were used in this study. These stimuli included presenting LEDs in the Hemifield and Quadrant field (Fig 1B). For Hemifield stimuli, all the LEDs on the hemi-half of the dome (left or right) were presented. Similarly the LEDs in a quadrant were presented for the Quadrant stimuli (upper right, upper left, lower right and lower left). The LEDs in the central 30 degrees were not presented in both these stimuli to avoid light scatter over to the other side. The LEDs were presented from a peripheral extent of 100 degrees on the left and right sides. In the inferior visual field, peripheral extent started from 60 degrees and superiorly from 100 degrees, because of the design of the dome.

### Experimental task

Infants were placed in a supine position on a mattress inside the Pediatric Perimeter (Fig 1A). The mother was kept by the infant’s side outside the perimeter with her hand constantly touching the infant’s feet to give reassurance. The testing was conducted by 2 experienced examiners (authors SD and PNS) on different days and was assisted by research assistants. Each infant was tested only once. This study was initiated as a part of a larger study to measure reaction time and visual field extent. Only the reaction time measurements are reported in this paper. Reaction time was computed from the time difference between the stimulus onset and the onset of the infant’s eye/head movement towards the stimuli direction (see *Video Analysis* below for further details).

Before starting the test, infant’s head position was centered to the infrared (IR) camera (HD USB camera (ELP-USBFHD01M, 640x480, 60fps)) that was mounted at the top of the dome. The IR camera recorded the entire testing procedure and this recorded video was used for off-line analysis. Infants were made to fixate at the fixation lights of the perimeter before starting a trial. To aid in the central fixation, colored LED stimuli patterns were presented to draw the infant’s attention to the center of the dome. Once centered, the colored patterns were turned off and simultaneously the fixation LEDs (luminance, 580nm) at the center of the dome were presented. If and when required the research assistant also positioned the infant’s head centrally. The testing started only after the infant fixated at the center. The six peripheral/gross visual field stimuli i.e. 2 hemifield + 4 quadrant field, were presented by the examiner in a random order. When possible, each stimulus was repeated at least twice. All the tests were carried out binocularly. Eye or head movement of the infant towards the peripheral stimuli was observed in real time (see S1 and S2 Videos) by the examiner. If a movement in the direction of the stimuli was detected, the examiner registered the response at which time the peripheral stimulus was turned off and the fixation target was turned on. The next trial began once the infant fixated centrally. The peripheral stimuli were presented till the infant looked towards the stimuli or for 10 seconds duration. This time duration was chosen based on our observations from an earlier study [1] that permitted enough viewing time.

### Video analysis

Post the testing, the recorded videos were analysed manually using Datavyu software version 1.0 (https://datavyu.org/). Three research assistants, naïve to the purpose of the study were randomly allotted with videos to analyse. The protocol of detecting eye/head movements from the video analysis were standardized and the research assistants were trained on sample videos before they analysed the actual videos. An intra-class correlation coefficient >0.80 was obtained indicating a strong agreement amidst the three research assistants.

Similar to the earlier study [1], the protocol for video frame analysis essentially looked for the infant’s eye/head movement towards the peripheral light stimuli, after the stimulus’s onset. If a movement was detected that trial was considered as valid, if not the trial was discarded. As per the protocol a trial was discarded if the infant moved their eye/head with the stimuli onset or just before the stimuli presentation or if they were not centered. Strict video analysis protocol was ensured to get a clean set of valid trials’ reaction time.

### Statistical analysis

The primary outcome from the video analysis was to calculate the reaction time. As reaction time data is usually non-normally distributed and the mean values can get skewed to extreme outlier values, non-parametric tests were chosen. The data obtained in this study was also non-normally distributed (One sample Kolmogorov Smrinov test, P<0.001). Wilcoxon Signed Rank test was used to compare the reaction time of the infants between the Hemifield and Quadrant stimuli with Bonferroni correction for multiple comparisons. Comparison across the age groups of the infants was done using the Kruskal-Wallis test and for post-hoc analysis Mann-Whitney U test was performed. Spearman rank correlation between reaction time as a function of age was also investigated.

## Results

A total of 188 parents were approached for participation, of which 28 refused and 160 parents (85% participation) enrolled in the study. From those enrolled, 30 infants were excluded, as 20 of them were preterm, 5 infants were more than 10 months of age, 3 infants were less than 3 months of age, 2 infants were diagnosed with ocular abnormalities. Additionally, 9 other infants were also excluded either because they were crying or fussy. Thus, 121 infants were tested and their data was included for the analysis. It was typical that some trials had to be discarded soon after initiating it because the infant was turning, moving or was not fixating at the center at the start of the trial. Nevertheless, valid testing was possible in all the 121 infants for some of the stimuli. Table 1 shows the total number of infants enrolled and those who contributed for the valid trials. In 24% (29 out of 121) of the infants all the stimuli were tested. A total of 861 trials were obtained. Within these trials reaction time greater than 5 seconds were discarded similar to the criteria used in a previous study [9]. This led to 3 trials getting discarded. Further, with stringent protocol followed during the off-line video analysis, few more trials were discarded. All this resulted in 35% of trials getting discarded and the remaining 65% trials were analyzed. See S1 Video (for a valid trial) and S2 Video (for a discarded trial).

**Table 1:** Distribution of infants tested.

Age group (months)	Total infants tested (male: female)	Median age (inter-quartile range) in months	Infants with valid responses
3–5	44 (18:26)	3.53 (3.45–4.01)	39
>5–7	30 (16:14)	6.08 (6.05–6.34)	28
>7–10	47 (29:18)	7.79 (7.18–9.11)	43

### Reaction time

Within each age group, no right and left differences were observed for both Hemifields (right and left) and Quadrants (upper right and upper left; lower right and lower left) stimuli (*P*>0.02, with Bonferroni correction for multiple comparisons). Hence a median reaction time combining the right and left stimuli was considered for further analysis.

**Fig 2:**
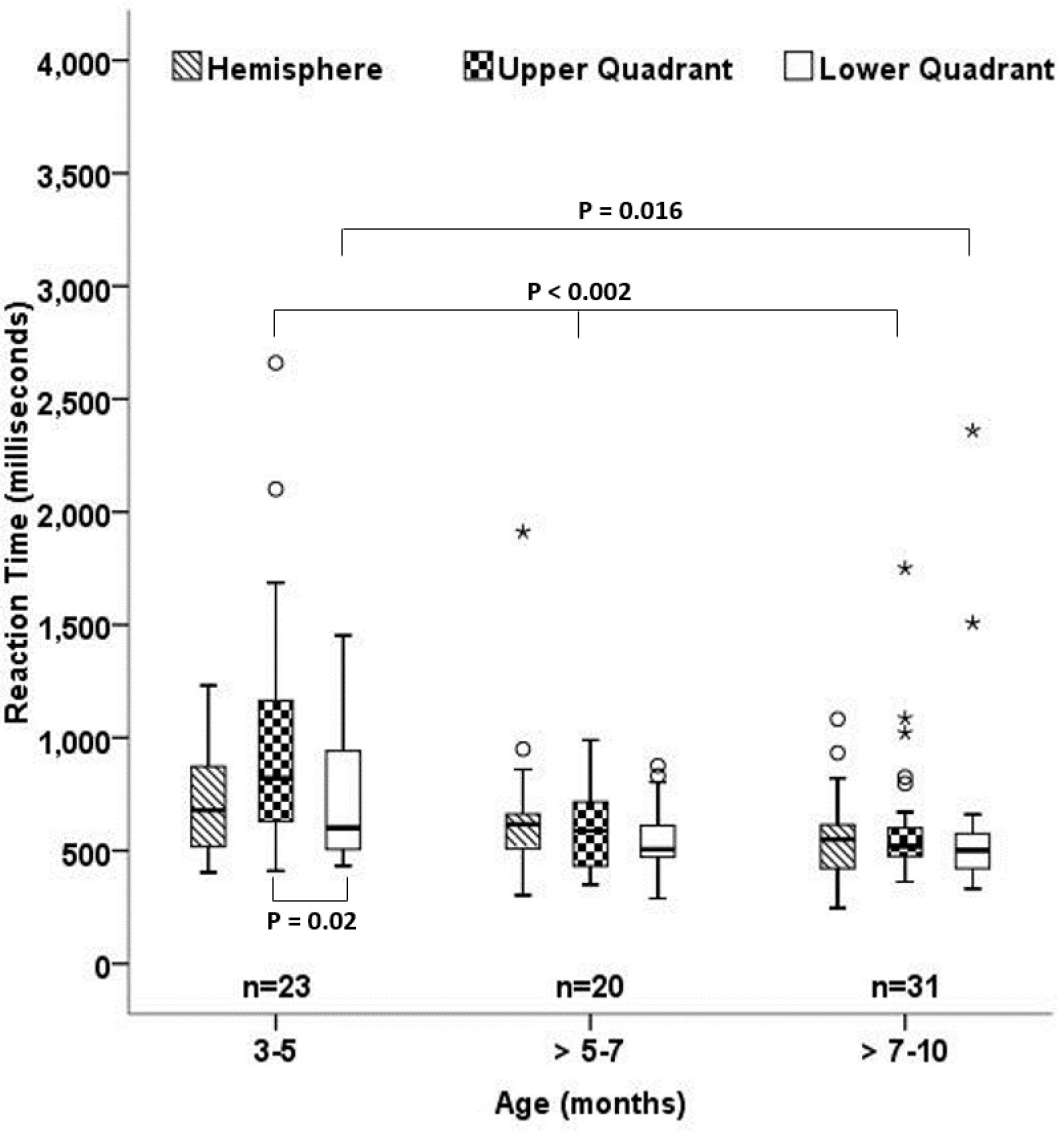
Box plot shows the reaction time measured in milliseconds for the 3 different age groups (x-axis) to the hemifields, upper quadrants and lower quadrants. The box denotes the interquartile range. Outliers (o) and extreme (*) values are also plotted.

No significant difference was observed between the three age groups (Kruskal-Wallis H(2) = 4.95, *P* = 0.084) for the Hemifield stimuli, although greater variability was observed in the younger 3–5 months age group (Fig 2). A significant difference was observed between the Upper and Lower Quadrants only for 3–5 months of age (Wilcoxon Signed Rank test, Z = -2.23, *P* = 0.025) and not in the other age groups (*P*>0.23). Tables 2 and 3 shows the median and interquartile range of the reaction time for the Hemifield and Quadrant stimuli. Reaction time was significantly longer for both the upper Quadrants (P<0.001) and lower Quadrants (P = 0.038) in the 3–5 months old infants when compared with the other two age groups. Upon post-hoc analysis, only the upper Quadrant was significantly different (Mann-Whitney U, P<0.002) for the 3–5 months age group when compared with both >5–7 and >7–10 age groups. The lower Quadrant stimulus was significantly different between 3–5 months and >7–10 months of age group (Mann-Whitney U, P = 0.016) and not with >5–7 months age group (Mann-Whitney U, P = 0.073). No significant difference was found for either Quadrants between the age groups >5–7 and >7–10 months (Mann-Whitney U, *P*>0.5). Fig 2 shows the reaction time distribution for all the stimuli in different age groups.

**Table 2:** Median reaction time to gross visual field stimuli.

Age Group (in months)	Median Reaction Time (Interquartile range) in milliseconds		
	Hemifield	Upper Quadrant	Lower Quadrant
3–5	587 (480–877)	820 (659–1093)	601 (540–1052)
>5–7	609 (487–661)	590 (452–741)	521 (470–629)
>7–10	550 (427–634)	534 (483–641)	504 (411–577)

**Table 3:** Median reaction time to all 4 quadrants for all the age group.

Age Group (in months)	Median Reaction Time (Interquartile range) in milliseconds			
	Left Upper Quadrant	Right Upper Quadrant	Left Lower Quadrant	Right Lower Quadrant
3–5	816 (601–1105)	830 (516–1091)	540 (441–920)	620 (479–863)
>5–7	580 (440–940)	607 (460–694)	558 (451–682)	545 (472–643)
>7–10	516 (409–600)	558 (461–688)	510 (392–624)	510 (411–597)

A linear regression was plotted for the upper quadrant as a function of age. The Spearman correlation coefficient showed a moderate negative correlation between the reaction time and age (R = -0.36, df = 98, P<0.001). A poor non-significant correlation (R = -0.13, df = 96, P = 0.1) was observed between lower quadrant and age. As the variability was large in these reaction times, a ratio between the upper and lower quadrants was calculated for each infant to negate individual variability. As this data was normally distributed a one-way ANOVA with Bonferroni post-hoc test showed that age group 3–5 months to be significantly (P = 0.027) different than >7–10 months age group and the other age group comparisons were not statistically significantly different.

## Discussion

With the Pediatric Perimeter, reaction time to gaze to peripheral stimuli was computed for infants in three different age groups and normative data has been documented. Similar to previous studies [10, 11] we hypothesized that the reaction time of older infants will be faster than younger infants. We were able to observe this trend and this was in agreement with earlier studies [4, 12]. There were three main findings from this study: younger infants had longer and more variance in reaction time, reaction time to quadrant stimuli were longer than hemifield stimuli and an asymmetry in reaction time between upper and lower quadrant stimuli disappears with increase in age. The implications of these findings are discussed below.

Disengaging and attending to a new target is known to be slower in younger infants [13]. We observed the variability (interquartile range) of the reaction time to decrease with age. However, there are few infants who are found to be outliers in all the three age groups (Fig 2). Attention factor could be an important reason for this variability. The reduction in reaction time along with its variability with age could also reflect the physiological reasons such as pruning of neural connections and maturation of the visual system [14].

While the trend of longer reaction time was seen in younger infants, a statistically significant reduction for reaction time with age was observed only for the stimuli shown in quadrants and not in hemifields when compared between the age groups. The lack of significance for the hemifield stimuli could be that some infants could have made a horizontal gaze to look while others could have made a vertical or oblique gaze. Quadrant stimuli could mostly elicit a vertical or oblique gaze response. Vertical saccadic eye movements are known to have longer latency than horizontal saccadic eye movements [15]. Such a trend was observed in this study for the younger infants with the quadrant stimuli. Suprathreshold stimuli (brighter and multiple LEDs) were used in this study unlike previous studies [16, 17] that showed only a single stimuli, hence sensitive differences may not have been picked up in this study.

Additionally a significant asymmetry in reaction time between the upper and lower quadrant stimuli only for infants in the 3–5 months of age (Fig 2) was observed. These infants were slower to look upwards than downwards by a factor of 1.4. In older infants this discrepancy disappears. It has been documented that infants tend to look more downwards than upwards [18]. It is unclear if more frequent downward saccades improve the efficiency of the saccadic system by reducing the latency [15]. Potential asymmetry in visual attention [19–22] could have also contributed to the vertical asymmetry in reaction time observed in this study. An asymmetry in maturation of the neural elements responsible for these gaze directions could also possibly explain this reaction time difference. Bilateral projections from burst neurons in the rostral interstitial nucleus of the medial longitudinal fasciculus is responsible for upward saccades and the ipsilateral projections from burst neurons to motoneurons are responsible for downward saccades [23]. Studies on vertical asymmetries in saccades for upper and lower targets have shown conflicting results (see Irving & lillakas [15] for review). These studies have been done on children and older adults.

The quadrant asymmetry observed in this study is interesting and can be explored further in infants having neurological conditions as well. In a previous pilot study [1], it was observed that children with developmental delay had a slower reaction time when compared to typically developing infants when viewing the stimuli presented in the Pediatric Perimeter. In that study however the overall reaction time to all stimuli was pooled together. If the vertical stimuli are sensitive to show a significant difference for maturation with age, such stimuli alone can be used for screening purpose and for long-term monitoring of neural development of the visual system. Further studies with stimuli specific to one meridian orientation (vertical or oblique) instead of a quadrant stimulus can be designed to sensitively pick up these differences. In the current clinical practice only qualitative observations for infant’s response to light are made e.g. “fixating and following light”. A quantitative measure as in this study could help for better management. For example fixation reaction time before and after surgery in infants having congenital cataract could be documented or longitudinal monitoring of the reaction time and comparing it with the normative data (Tables 2 and 3) in managing infants who have delayed visual maturation is also possible.

Both eye and head movements were allowed in our experimental set up. It is known that by the age of 3 months onwards infants can develop coordinated eye-head movements [24, 25] and make directionally appropriate saccades [17]. The greater variability in the younger age group could also be indicative of developmental differences for the eye-head coordination between the infants. Given this variability, a larger sample size within each age group will be needed for future studies. With the absence of an eye/head tracker it could be possible that a human observer could have missed detecting a small eye/head movement in real time. Since the video recordings were analyzed offline and assessed for valid trials, this limitation was minimized. The manual analysis of videos can be time consuming, future directions in this work aims to use automated or semi-automated algorithms for detection.

In conclusion, reaction time of infant’s eye/head movements to peripheral stimuli can be measured using the Pediatric Perimeter device. Reaction time was found to reduce with age. The reduction is significant for the upward gaze movements. The asymmetry in reaction time between the upper and lower quadrant stimuli also reduces with age. The reduction in these parameters could indicate maturity with age for the neural elements generating these gaze movements. Visual attention could also play a role. The data obtained in this study provides a reference normative value for gaze reaction time for different age groups of infants. Such a reference criteria can refine the clinical examination of infants. Further studies can be undertaken to understand the diagnostic use of this parameter in several neurological conditions that can affect the visual system in children.

## Supporting information

S1 Video Video of a 9-month old infant getting tested in the Pediatric Perimeter. It can be seen that the infant is aligned to the center before the presentation of the stimuli. This video is a representative video of trials being labeled valid and included for the analysis.
(MP4)

S2 Video Video of a 9-month old infant getting tested in the Pediatric Perimeter. It can be seen that the infant was not aligned to the center when the stimuli was presented. This video is a representative video for discarded trials due to poor centration.
(MP4)

S1 Data (XLSX)
